# RNA sequencing identifies novel non-coding RNA and exon-specific effects associated with cigarette smoking

**DOI:** 10.1186/s12920-017-0295-9

**Published:** 2017-10-06

**Authors:** Margaret M. Parker, Robert P. Chase, Andrew Lamb, Alejandro Reyes, Aabida Saferali, Jeong H. Yun, Blanca E. Himes, Edwin K. Silverman, Craig P. Hersh, Peter J. Castaldi

**Affiliations:** 1 Channing Division of Network Medicine, Brigham and Women’s Hospital, 181 Longwood Ave, Boston, MA USA; 2 Harvard Medical School, Boston, MA 02115 USA; 3 Department of Biostatistics and Computational Biology, Dana-Farber Cancer Institute, Boston, MA USA; 4 Division of Pulmonary and Critical Care Medicine, Brigham and Women’s Hospital, Boston, MA USA; 5 Department of Biostatistics, Epidemiology, and Informatics, Perelman School of Medicine, University of Pennsylvania, Philadelphia, PA USA; 6 Division of General Internal Medicine and Primary Care, Brigham and Women’s Hospital, Boston, MA USA

**Keywords:** RNA-seq, Differential expression, Cigarette smoking, Exon usage, Isoforms

## Abstract

**Background:**

Cigarette smoking is the leading modifiable risk factor for disease and death worldwide. Previous studies quantifying gene-level expression have documented the effect of smoking on mRNA levels. Using RNA sequencing, it is possible to analyze the impact of smoking on complex regulatory phenomena (e.g. alternative splicing, differential isoform usage) leading to a more detailed understanding of the biology underlying smoking-related disease.

**Methods:**

We used whole-blood RNA sequencing to describe gene and exon-level expression differences between 229 current and 286 former smokers in the COPDGene study. We performed differential gene expression and differential exon usage analyses using the voom/limma and DEXseq R packages. Samples from current and former smokers were compared while controlling for age, gender, race, lifetime smoke exposure, cell counts, and technical covariates.

**Results:**

At an adjusted *p*-value <0.05, 171 genes were differentially expressed between current and former smokers. Differentially expressed genes included 7 long non-coding RNAs that have not been previously associated with smoking: *LINC00599, LINC01362, LINC00824, LINC01624, RP11-563D10.1, RP11-98G13.1, AC004791.2*. Secondary analysis of acute smoking (having smoked within 2-h) revealed 5 of the 171 smoking genes demonstrated an acute response above the baseline effect of chronic smoking. Exon-level analyses identified 9 exons from 8 genes with significant differential usage by smoking status, suggesting smoking-induced changes in isoform expression.

**Conclusions:**

Transcriptomic changes at the gene and exon levels from whole blood can refine our understanding of the molecular mechanisms underlying the response to smoking.

**Electronic supplementary material:**

The online version of this article (10.1186/s12920-017-0295-9) contains supplementary material, which is available to authorized users.

## Background

Cigarette smoking is the leading modifiable risk factor for disease and death worldwide. In the United States, smoking accounts for more than 438,000 deaths per year [1], and since 1964 more than 20 million Americans have died because of smoking [2]. Cigarette smoking increases risk of many diseases including cancer, chronic obstructive pulmonary disease, coronary heart disease, and stroke [3]. However, research shows smoking cessation can reduce risk of many diseases, in some cases reverting disease risk to the level of non-smokers [4, 5]. This suggests specific molecular changes occur in active smoking (as compared to former smoking) that increase disease risk.

To identify the molecular mechanisms underlying response to smoke exposure, previous studies have characterized gene expression changes in a number of human tissues including, peripheral whole blood [6–9], lymphocytes [10], monocytes [11], bronchial epithelial cells [12, 13], alveolar macrophages [14], and lung tissue [15–17]. This includes a recently published meta-analysis of 1421 current, 3955 former, and 4860 never smokers that identified 1270 differentially expressed genes between current and never smokers and 39 differentially expressed genes between former and never smokers in peripheral blood [6]. These results focused on gene level quantification from microarrays. However, alternative splicing and differential isoform usage play a critical role in human biology, but little is known about alternative splicing with respect to cigarette smoking.

RNA sequencing (RNA-seq) facilitates the ability to look at more complex regulatory phenomena such as isoform-switching, alternative promoter usage, and exon inclusion/exclusion events. Moreover, it can interrogate not only known mRNA transcripts, but additional populations of RNA including long non-coding RNAs (lncRNAs), small RNAs and microRNAs. We hypothesized that: 1) RNA-seq of peripheral blood from smokers could refine our understanding of the molecular mechanisms underlying the response to cigarette smoking; and 2) that some transcripts show an acute response to smoke exposure above and beyond the chronic changes. We sought to answer these questions by performing gene-level differential expression and differential exon usage (DEU) analysis in 515 current and former smokers from the COPDGene study [18], a large, well-characterized cohort that included both Non-Hispanic White and African American participants.

## Methods

### Study participants

Our study included 515 participants of the COPDGene study. A complete study protocol for COPDGene had been described elsewhere [18], but briefly, self-identified Non-Hispanic Whites and African Americans between the ages of 45 and 80 years with a minimum of 10 pack-years lifetime smoking history (1 pack-year = 1 pack of cigarettes smoked daily for 1 year) were enrolled at 21 centers across the United States. Subjects returned for a second study visit approximately 5 years after initial enrollment, at which point they completed detailed questionnaires, pre- and post- bronchodilator spirometry, volumetric computed tomography of the chest, and provided blood for complete blood counts (CBCs) with differentials and RNA sequencing Subjects were cancer-free at time of study enrollment.

Smoking history was ascertained by self-report. Participants defined as current smokers answered yes to the question “Do you smoke cigarettes now (as of one month ago?)”. Participants defined as acute smokers answered yes to the question “Have you smoked a cigarette(s) in the past 2 hours?”. Sequenced subjects included COPD cases (GOLD spriometric stage 2,3 or 4 [19]) and smokers with normal lung function (GOLD stage 0 or 1) with available chest computed tomography. Institutional review board approval and written informed consent was obtained for all subjects.

### RNA extraction

Total RNA was extracted from PAXgene ™ Blood RNA tubes using the Qiagen PreAnalytiX PAXgene Blood miRNA Kit (Qiagen, Valencia, CA). The extraction protocol was performed either manually or with the Qiagen QIAcube extraction robot according to the company’s standard operating procedure. Extracted RNA samples with RIN > 7 and concentration > =25 μg/ul were sequenced.

### cDNA library preparation and sequencing

Globin reduction and cDNA library preparation for total RNA was performed with the Illumina TruSeq Stranded Total RNA with Ribo-Zero Globin kit (Illumina, Inc., San Diego, CA). Libraries were QCed by quantification with picogreen, size analysis on an Agilent Bioanalyzer or Tapestation 2200 (Agilent, Santa Clara, CA) and qPCR quantitation against a standard curve. 75 bp paired end reads were generated on a HiSeq 2500 flow cell. Libraries are loaded at an empirically determined concentration in order to generate the optimal number of clusters per lane of the flow cell. Samples were sequenced to an average depth of 20 million reads.

### Read alignment, expression quantification, and sequencing quality control

Reads were trimmed of TruSeq adapters using Skewer with default parameters [20]. Trimmed reads were aligned to the GRCH38 genome using the STAR aligner version 2.4.0 h [21]. Gene and exon level counts were generated using RSubreads [22] with the Ensembl version 81 annotation [23]. Quality control was performed using the FastQC [24] and RNA-SeQC programs [25]. Samples were included for subsequent analysis if they had >10 million total reads, >80% of reads mapped to the reference genome, *XIST* and Y chromosome expression was consistent with reported gender, <10% of R1 reads in the sense orientation, Pearson correlation > = 0.9 with samples in the same library construction batch, and concordant genotype calls between variants called from RNA sequencing reads and DNA genotyping. The gene count data used for this analysis are available in GEO [26, 27] (accession number GSE9753).

### Technical covariates

To remove unwanted batch effects and confounders, we applied SVAseq [28] to the gene or exon count matrices. Surrogate variables (SVs) were estimated while specifying the following covariates: age, gender, race, pack-years of smoking history, library construction batch and cell count percentages.

### Gene-level differential expression analyses

We performed differential gene expression analysis using the voom [29] /limma [30, 31] R package. Transcripts that were expressed at > = 1 count per million mapped reads in > = 10 subjects were analyzed. Analyses compared current and former smokers controlling for age, gender, race, pack-years of smoking history, monocyte percentage, lymphocyte percentage, eosinophil percentage, neutrophil percentage, library construction batch, and significant SVs (*n* = 27). Differentially expressed genes were defined with as those with an empirical Bayes corrected *p*-value <0.05.

To assess if differentially expressed genes were associated with acute smoking, we performed differential gene expression in limma [30, 31] comparing current smokers who had smoked within the past 2 h to current smokers who had not (controlling for age, gender, race, pack-years of smoking history, cell count percentages, library construction batch, and significant SVs [*n* = 14]). Follow-up sensitivity analysis additionally controlled for the average number of cigarettes smoked per day to test if differential expression results could be explained by smoking intensity. Genes were considered significant if their Bonferroni corrected *p*-value was <0.05 (corrected for the number of differentially expressed genes).

### Gene ontology (GO) enrichment analyses

To identify gene sets over or under-represented in differentially expressed genes, we performed GO gene ontology enrichment analyses using PANTHER (accessed through: http://www.geneontology.org) [32–34]. Analysis input included all significantly differentially expressed genes, and queries included gene sets in the “biological processes” ontology (database version released 2017–01-26). Significant gene sets were defined as those with a Bonferroni corrected *p* value <0.05.

### Differential exon usage analyses

We tested for DEU between current and former smokers using the topSplice function within the limma R package [30, 31]. Adjusted *p*-values less than 0.05 in topSplice were confirmed using the DEXseq R package (alternate version, accessed through github/areyesq89/DEXSeqAlt) [35]. In contrast with the original version, this alternate version uses the statmod GLM fitter and skips the step of sharing information across exons when calculating dispersion estimates, which is not needed for analysis of large sample sizes. Both analyses were performed using exon level counts generated by Rsubreads. TopSplice uses a moderated T-statistic to test for differences between each exon and all other exons for the same gene, while DEXseq tests a full GLM with an exon x condition interaction term (~sample + exon + exon:smoking + exon:covariates) versus a reduced GLM without an exon x condition interaction term (~sample + exon + exon:covariates) via a likelihood ratio test. Therefore, both approaches explicitly test for DEU between current and former smokers while accounting for differences in overall gene expression. Exons with a topSplice adjusted *p*-value <0.05 and a DEXseq p-value <0.05 were defined as DEU.

## Results

### Demographics

A total of 229 current and 286 former smokers were included in our analysis. All subjects had a substantial smoking history (mean pack-years smoked = 45) with current smokers more likely to be younger and African American. As expected, smoking was associated with changes in peripheral blood cell counts with current smokers having significantly lower neutrophil and monocyte percentages and a higher lymphocyte percentage (Table 1).

**Table 1:** Summary of analyzed COPDGene subjects by former (*n* = 286) and current (*n* = 229) smoking status. Values represent mean (SD)

	Former Smokers (n = 286)	Current Smokers (n = 229)	*P*-value
Race (% NHW)	86%	57%	<0.01
Gender (% female)	45%	49%	0.4
Age	69 (8.1)	61 (7.6)	<0.01
Pack-Years Smoked	44 (23)	46 (21)	0.5
FEV_1_ percent predicted	73 (29)	79 (24)	0.02
COPD cases	41%	48%	<0.01
Neutrophil percentage	61 (11)	58 (11)	<0.01
Lymphocyte percentage	28 (11)	31 (9.9)	<0.01
Eosinophil percentage	2.6 (2.1)	2.7 (3.0)	0.9
Monocyte percentage	8.5(2.5)	7.6(2.2)	<0.01
Basophil percentage	0.59(0.55)	0.59(0.65)	0.9

### Differential gene expression in response to cigarette smoke

**Fig. 1:**
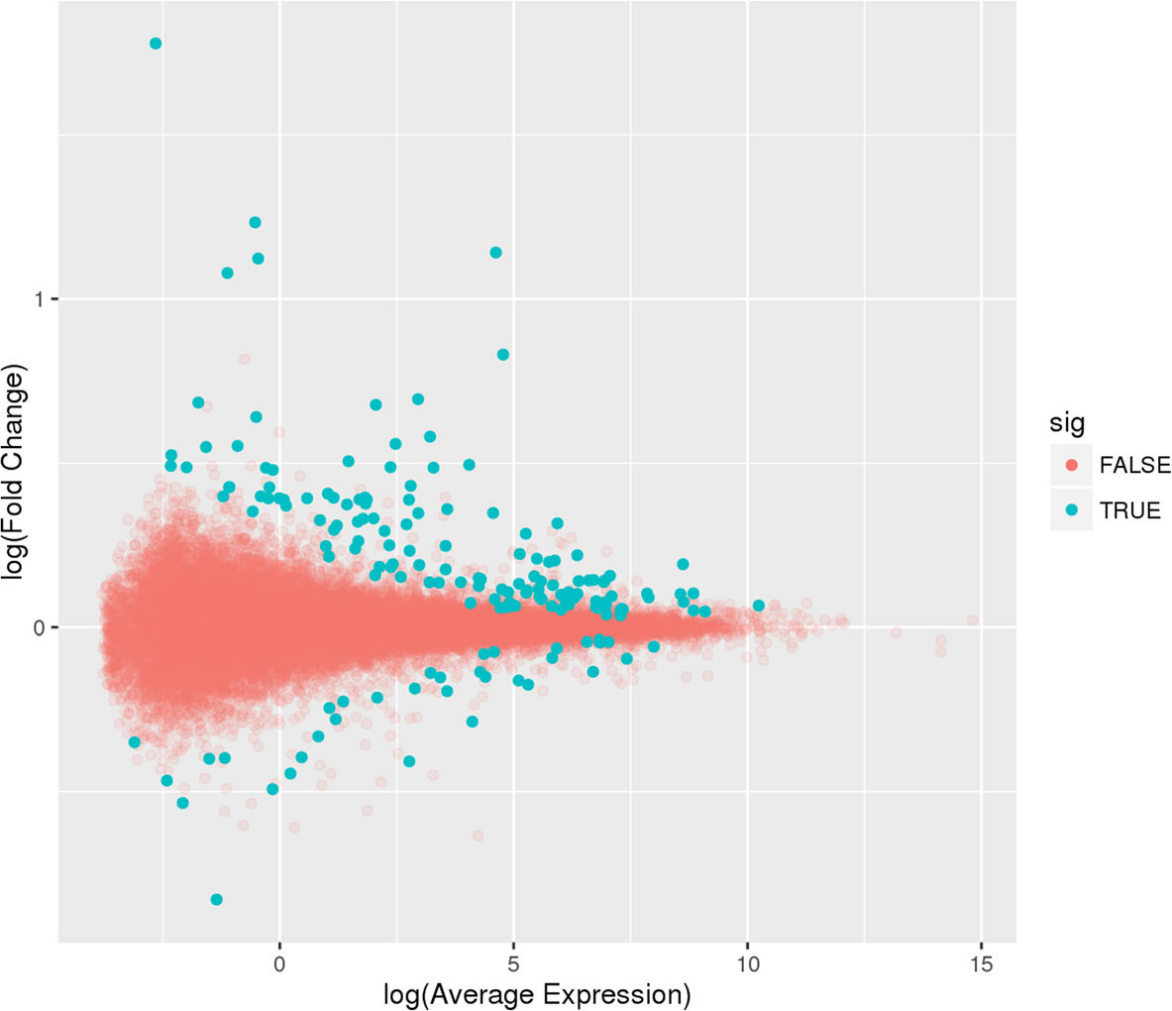
Mean-Average(MA)-plot of log2 average expression versus log2 fold change between current and former smokers. Log fold change values greater than zero indicate genes with higher expression in current smokers (n = 136 genes), Log fold change values less than zero indicate genes with higher expression in former smokers (*n* = 35 genes). Blue dots indicate genes that are significantly differentially expressed (adjusted *p* value <0.05)

We observed 27,885 expressed genes, including 14,866 protein coding genes, 3277 processed pseudogenes and 2204 lncRNAs (Additional file 1: Figure S1). At an adjusted *p*-value <0.05, we identified a total of 171 differentially expressed genes between current and former smokers (Additional file 2: Table S1), the majority of which (79.5%) had higher expression in smokers (Figure 1). Effect sizes of differentially expressed genes ranged from −0.83 to 1.78, with 5 of 171 having a log_2_ fold change greater than 1.0 (*SEMA6B*, *AHRR*, *GPR15*, *CTTNBP2*, and *LINC00599*). Significant results were enriched for genes previously identified by a large microarray expression study of current versus never smokers [6] (50 of 171 genes overlap, *p*-value hypergeometric test of upregulated genes <0.001, p-value hypergeometric test of downregulated genes <0.001), with the direction of effect being consistent in all 50 overlapping genes (Additional file 3: Table S2). The top 2 differentially expressed genes, *GPR15* and *LRRN3,* have been previously reported in both expression [6, 7] and methylation studies of smoking [36–40].

Included in the differentially expressed genes were 7 lncRNAs that have not been previously associated to smoking (*LINC00599, LINC01362, LINC00824, LINC01624, RP11-563D10.1, RP11-98G13.1, and AC004791.2*). Interestingly, 6 of the 7 have higher expression in current smokers as compared to former smokers, suggesting an up-regulation of lncRNAs in response to cigarette smoking (Table 2, Additional file 4: Figure S2). The gene with the largest effect size, *LINC00599*, showed minimal expression in former smokers (mean normalized count = 0.1) but had a marked increase in current smokers (mean normalized count = 0.9), with 91% of observations in the top quintile of expression being current smokers (Additional file 5: Figure S3). To test if significant lncRNA findings were represented in previous microarray studies, we cross-referenced our 7 significant findings with the maps from the Illumina Human HT12 versions 3 and 4 microarrays. Probes mapping to LINC00824 and *RP11-98G13.1* were present on the Illumina Human HT12 version 3 array, but none of the 7 significant findings were present on version 4.

**Table 2:** Differentially expressed long non-coding RNAs (lncRNA) between current and former smokers (adjusted p-value <0.05). Of the 7 differentially expressed lncRNAs, 6 have higher expression in current smokers as compared to former smokers

Ensembl Gene ID	Gene Symbol(s)	Chr	Log Fold Change	Average Expression	Moderated T Statistic	P Value	Adjusted P Value
ENSG00000253230	LINC00599,RNCR3	8	1.777	−2.655	11.439	5.87E-27	5.46E-23
ENSG00000227240	RP11-563D10.1	1	0.426	−0.222	4.912	1.24E-06	7.52E-04
ENSG00000230817	LINC01362	1	0.553	−0.903	4.319	1.91E-05	6.99E-03
ENSG00000237011	RP11-98G13.1	1	0.399	−0.408	4.099	4.88E-05	1.49E-02
ENSG00000267453	AC004791.2	19	0.685	−1.743	4.021	6.72E-05	1.94E-02
ENSG00000254275	LINC00824, LINC01263	8	0.407	1.028	4.013	6.96E-05	1.98E-02
ENSG00000227508	LINC01624,TCONS_00011425	6	−0.225	1.356	−3.948	9.07E-05	2.22E-02

To assess if time since smoking cessation modified our results, we performed differential expression analysis of this quantitative outcome in former smokers with this measurement available (*n* = 270). Mean time since smoking cessation in former smokers was 17.3 years (sd = 10.86). There was one significantly differentially expressed gene associated with this outcome (*GPR15*, adjusted *p*-value = 2.9 × 10^−8^).

### Gene ontology (GO) enrichment analysis

GO functional enrichment analyses identified 41 biological pathways significantly over-represented and no pathways under-represented in the 171 differentially expressed genes at a Bonferroni corrected p-value <0.05 (Additional file 6: Table S3). The most significant GO gene sets included “immune system process” (GO:0002376, adjusted *p* value = 1.96 × 10^−7^), “defense response” (GO:0006952, adjusted p value = 6.26 × 10^−7^), and “response to external stimulus” (GO:0009605), adjusted *p* value = 6.60 × 10^−5^).

### Transcriptomic response to acute smoking

To assess if the 171 differentially expressed genes were associated with acute smoking, we performed gene-based limma analysis comparing smokers who had smoked at least 1 cigarette within the past 2 h (*n* = 93) to those who had not (*n* = 136). Five genes were significantly differentially expressed (Bonferroni corrected for 171 tests), and 29 of 171 had a nominal *p*-value <0.05 (Additional file 7: Table S4). When considering all expressed transcripts, none were significantly differentially expressed with acute smoking at an adjusted *p*-value <0.05.

**Fig. 2:**
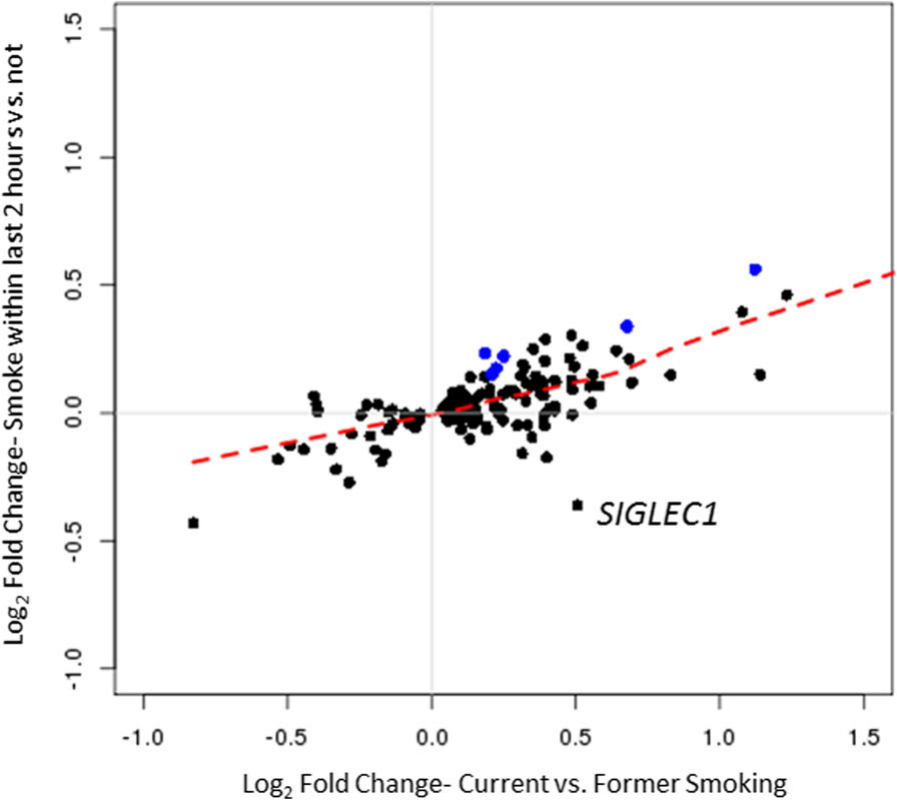
Comparison of effect sizes between differential expression analyses of: 1) current versus former smoking (defined as smoking cigarettes within the past month) on the x-axis and 2) acute smoking (defined as smoking cigarettes within the past 2 h) on the y-axis. Each point represents a differentially expressed gene in the current smoking analysis (n = 171). Blue dots represent 6 genes significantly differentially expressed between those who have smoked within the past 2 h and those who have not (Bonferroni corrected for 171 genes). The red line is a fitted via lowess smoothing. The labeled gene (*SIGLEC1)* shows an opposite direction of effect between the 2 analyses (logFC current smoking = 0.51, logFC acute smoking = −0.36)

Overall, there was a significant correlation between the fold changes calculated in the current smoking and acute smoking analyses (Pearson = 0.70, *p*-value <0.001, Figure 2), however some genes had an opposite direction of effect between the 2 analyses (e.g. *SIGLEC1*, log_2_ fold change current smoking = 0.51, log_2_ fold change acute smoking = − 0.34). Sensitivity analysis controlling for smoking intensity (measured as the average number of cigarettes smoked per day) yielded similar results (Additional file 7: Table S4). This suggests that some of the 171 smoking genes demonstrate an acute response to smoking exposure above and beyond the baseline effect of chronic smoking, whereas others do not. GO enrichment on the 29 nominally significant genes revealed the top annotation as “chemotaxis” (Bonferroni adjusted *p* value = 0.08), suggesting that there may be an effect of acute smoke exposure on cell signaling and migration in chronic smokers.

### Differential exon usage

We used complementary methods (limma’s topSplice and DEXseq) to test for differential exon usage (DEU) between current and former smokers. A total of 119,217 exons had expression levels suitable for DEU analysis. Exon-level *p*-values showed no evidence of systematic inflation (Additional file 8: Figure S4).

**Fig. 3:**
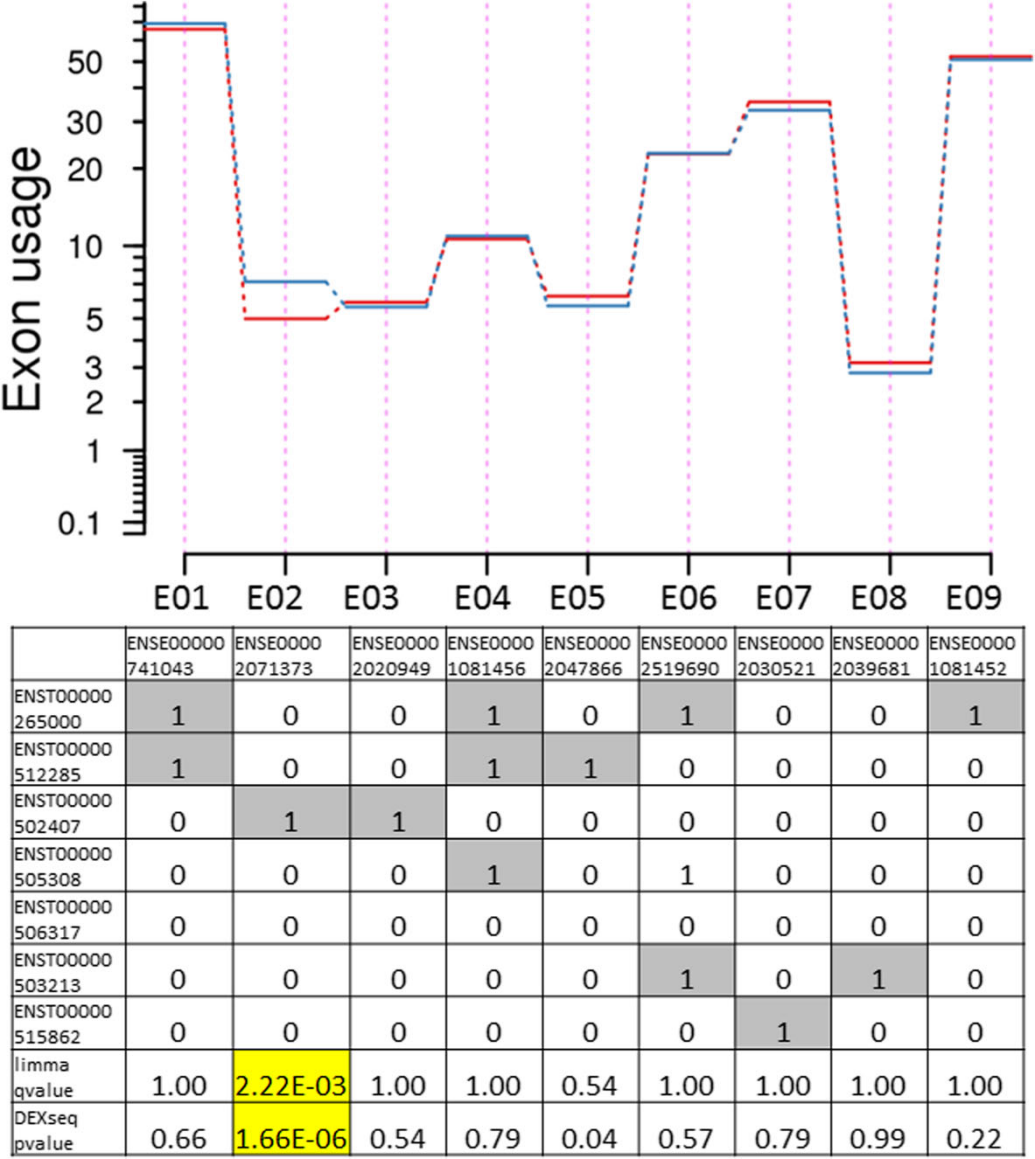
Exon usage in the *GALNT7* gene by smoking status. The top plot shows exon usage for each analyzed exon by smoking status (red = former, blue = current). One exon showed significant differential usage between current and former smokers (ENSE00002071373). The bottom table maps tested exons to known isoforms (1 = exon present in that transcript, 0 = exon not present in that transcript)

In total, 9 exons in 8 genes showed significant DEU (Table 3, Additional files 9, 10, 11, 12, 13, 14, 15: Figures S5-S11). Although all genes with DEU had multiple isoforms (range 4–17), 8 of 9 significant exons were annotated to only one isoform, suggesting that the identified DEU exons tag isoform differences between current and former smokers (Fig. 3). Significant exons were most likely to be the last exon of a transcript (5/9) or the first exon of a transcript (3/9) and one significant DEU exon (in *MANIA1*) was located in the middle of its associated transcript.

**Table 3:** Differential exon usage (DEU) between current and former smokers. Significant DEU is defined as an adjusted *p*-value <0.05 from limma exon-based T statistic and DEXseq p-value <0.05.Gene-based P value is unadjusted from limma differential expression analysis

Ensembl Exon ID	Gene Symbol	Limma Adjusted *P* value	DEXseq *P* value	Gene-based *P* value	Transcripts Containing Exon	Exon Number in Transcript
ENSE00001810132	EPS15	4.00E-02	2.00E-02	8.16E-01	ENST00000478657	first
ENSE00002071373	GALNT7	2.22E-03	1.66E-06	1.30E-01	ENST00000502407	first
ENSE00001444573	SASH1^a^	1.80E-09	2.39E-03	4.22E-21	ENST00000367467	last
ENSE00001400828	AREL1	1.27E-02	1.18E-03	9.98E-01	ENST00000356357	last
ENSE00001444981	UTRN	5.72E-16	8.82E-02	1.75E-01	ENST00000367545	last
ENSE00001635177	MAN1A2	4.00E-03	3.25E-02	9.48E-03	ENST00000356554	second
ENSE00001231507	LRRN3^a^	3.23E-10	4.69E-04	4.19E-36	ENST00000308478	first
ENSE00001175333	ERAP1	7.87E-12	1.27E-03	4.27E-01	ENST00000296754	first
ENSE00001641703	ERAP1	1.19E-06	3.63E-03	4.27E-01	ENST00000443439	first

^a^Differentially expressed gene

Of the 171 DE genes, only 2 showed significant DEU. Even after relaxing the significance level of the DEU analysis to *p* < 0.05, only 18 of 171 DE genes showed nominally significant DEU. Conversely, only 3 of the 9 exons identified in the DEU analysis showed nominal significance in the gene-based analysis (Table 3). This suggests some of the transcriptomic changes that occur in response to smoking are independent of differential gene expression and occur at the level of RNA processing. Gene and exon level results can be viewed interactively at https://cdnm.shinyapps.io/Current_smoking_Limma/.

## Discussion

By analyzing RNA-seq data from peripheral blood samples of 515 COPDGene study subjects, we identified 171 differentially expressed transcripts between current and former smokers, including 7 novel lncRNAs. Secondary analysis of 2-h smoking in current smokers showed that the majority of these 171 genes demonstrate a consistent, ongoing response to smoking while a subset of these genes fluctuate in response to acute exposure. Additionally, DEU analysis identified 9 differentially used exons between current and former smokers, suggesting smoking-induced changes in isoform expression.

Included in the 171 differentially expressed transcripts are 7 lncRNAs. LncRNAs are an abundant class of RNA defined by their length (> 200 base pairs) and the fact that they do not code for a protein [41]. Their function is largely uncharacterized, but they are thought to broadly regulate transcription through multiple mechanisms including [42, 43]: 1) chromatin remodeling (lncRNAs can affect the recruitment of polycomb repressive complexes that modify histones to cause gene silencing) [44, 45]; 2) transcriptional co-factors (e.g. the most abundant gene in our data [*MALAT1*] is a lncRNA that acts as a cofactor to increase or decrease expression of proximal genes) [46, 47]; and 3) competition for endogenous RNAs (i.e. lncRNAs can act as a sponge for microRNAs thereby inhibiting their effect) [48, 49]. For example, *LINC00599* (the most significant differentially expressed lncRNA between current and former smokers) is hypothesized to regulate transcription by competing for shared microRNAs. Previous research has associated this transcript with atherosclerosis-related vascular dysfunction [50]. Authors identified 3 microRNA binding sites on *LINC00599* (hsa-miR-4306, hsa-miR-185-5P, hsa-miR-4644), proposing that increased expression of *LINC00599* causes a decreased concentration of these microRNAs with corresponding alterations in the abundance of their mRNA targets. To test this, we looked for enrichment of our differentially expressed genes in these predicted targets (*n* = 467 in the StarBase database [51] and *n* = 373 in the TargetScan database [52]). However, we did not find significant overlap (hypergeometric *p*-value for enrichment in StarBase = 0.15, hypergeometric p-value for enrichment in TargetScan = 0.29.)

We found evidence for differential exon usage in 9 exons from 8 genes. Interestingly, 8 of these 9 were unique to one transcript, suggesting these results may tag isoform differences between current and former smokers. Even in the case of *ERAP1* (the only gene with more than one differentially used exon), the 2 identified exons were unique to a single transcript (Additional file 11: Figure S7). Of note, 5 genes with significant DEU showed no evidence of differential expression in the gene-based analysis (unadjusted p-value >0.05). This suggests that some transcriptomic changes happen only at the level of RNA processing and do not affect mean gene expression levels. These findings highlight the potential utility of differential exon usage to identify potential isoform-specific effects, particularly with the challenges in accurately inferring isoform abundance from short-read RNA-seq data.

In 8 of the 9 instances of DEU, the involved exon was either the first or last exon in a transcript. There are a number of potential explanations for this finding. First and last exons tend to be larger than other exons, so it is possible that these results reflect increased statistical power relative to shorter exons. Alternatively, first and last exons play key roles in the initiation and termination of transcription. A recent analysis of GTEx data identified alternative transcription start and stop sites as the driving factor in differential exon usage across tissues [53]. Activating histone modifications (H3K4me3 and H3K9ac) map to the first exon-intron boundary and are known determinants of transcription quantity, transcription start site, and gene activity [54]. Last exons play an important role in transcription termination, and differential exon usage in the last exon may indicate 3′ UTR shortening or early transcription termination. In addition, mammalian transcription elongation is highly regulated and related to splicing [55]. Since our total RNA isolation methods include nuclear and partially processed RNAs, the concentration of DEU in first and last exons in our data may reflect smoking-related, gene-specific alterations in transcription initiation, elongation, or termination.

This study has a number of strengths: to our knowledge, this is the first large-scale RNA-seq analysis of cigarette smoking, and it is the first study to describe differential exon usage between current and former smokers. RNA-seq allows for the unbiased identification of novel differentially expressed transcripts, and this study identified novel associations with smoking and seven lncRNAs. Additionally, although cigarette smoking was associated with changes in total peripheral cell counts, all subjects had measured blood cell counts (CBCs) at the time of RNA sequencing. This allowed for direct adjustment of cell-specific effects, mitigating against the possibility that results are due to cell type proportion confounding. We also used surrogate variable analysis to adjust for unmeasured confounders, including unmeasured cell type subpopulations.

Our study also has a number of limitations. We measured transcript expression in whole blood samples, thus our findings are most relevant to smoking-related alterations in circulating immune cells. While immune function is linked to a broad range of diseases, there are many other tissue-specific transcriptomic effects of smoking that are not captured in this study. Whole blood is a mixture of cell types, and while we were able to adjust for measured cell counts our differential expression results cannot pinpoint cell-type specific expression differences and residual confounding by unmeasured cell subpopulations is possible. It is possible that some of the differentially expressed genes or differentially used exons from this analysis may reflect alterations in unmeasured cell types. Future work in isolated cell populations will be needed to relate these observations to the specific cell types in which these transcriptomic changes occur, providing important validation and functional elucidation of these observations. Secondly, our outcomes of interest (current smoking and 2 h smoking) were based on self-report using a validated questionnaire [56] without biochemical confirmation, and may not completely capture the toxic effects of tobacco. Additionally, our samples were sequenced to an average depth of 20 million reads. While this depth provides good resolution for highly expressed transcripts, deeper sequencing would likely reveal differences in lower expressed features, including exons and isoforms. Finally, we focused on differential exon usage instead of isoform level analysis, because quantification of isoform abundance from short read data is still a significant challenge. Isoform inference algorithms including RSEM [57], kallisto [58], and salmon [59] have made significant improvements in isoform quantification, but performance is not yet at a level where differential isoform expression can be reliably detected [60].

## Conclusions

We used RNA-seq in a large study of current and former smokers to identify transcripts that are altered by smoking (via differential expression or differential exon usage). Our results suggest that there is an overall up-regulation of genes expressed in response to smoking, including an up-regulation of lncRNAs. These analyses provide the first exon-level observations of transcriptomic alterations induced by cigarette smoking in blood. Additional analysis in pure cell types isolated from current and former smokers is needed to understand the consequences of these changes on transcriptional networks and downstream processes. The gene and exon-level effects observed in this study refine our understanding of the molecular mechanisms underlying the response to cigarette smoking.

## Additional files

Additional file 1: Figure S1. Gene annotation of 27,885 observed genes (Ensembl version 81 annotation). (PNG 19 kb)

Additional file 2: Table S1. Genes differentially expressed (adjusted *p*-value <0.05) between current and former smokers (*n* = 171). Analysis adjusted for age, race, gender, pack-years of smoking history, monocyte percentage, lymphocyte percentage, eosinophil percentage, neutrophil percentage, library construction batch, and surrogate variables. (XLSX 28 kb)

Additional file 3: Table S2. Comparison of log fold changes in 50 differentially expressed genes that overlap Huan et al. 2016. All 50 genes have a consistent direction of effect. (XLSX 11 kb)

Additional file 4: Figure S2. Quantile-quantile (QQ) plots for differential gene expression analysis between current and former smokers using voom/limma. (PNG 14 kb)

Additional file 5: Figure S3. Normalized counts in current versus former smokers for lncRNAs that are significantly differentially expressed: A) *ENSG00000253230*; B) *ENSG00000227240*; C) *ENSG00000230817*; D) *ENSG00000237011*; E) *ENSG00000267453*; F) *ENSG00000254275*; G) *ENSG00000227508*. In 6 of 7 differentially expressed lncRNAs, current smokers have higher expression than former smokers**.** Y-axis represents log_2_ expression level. (PNG 118 kb)

Additional file 6: Table S3. Significant gene ontology terms over-represented in genes differentially expressed between current and former smokers. *P*-values are Bonferroni corrected for multiple comparisons. (XLSX 16 kb)

Additional file 7: Table S4. Differentially expressed genes in current versus former smokers (adjusted p-value <0.05) that are nominally significant (*p* < 0.05) in differential expression analysis of current smokers who had smoked within the last 2 h (*n* = 93) versus those who had not (*n* = 136). A total of 34 genes (20.5%) showed nominal significance in the acute smoking analysis. Secondary analysis adjusted for smoking intensity (i.e. average number of cigarettes smoked per day) yielded similar results. logFC = log_2_ fold change. AS = acute smoking. CS = current smoking. * = Adjusted for smoking intensity (average cigarettes per day). Bolded *p*-values are statistically significant after Bonferroni correction for 171 tests (XLSX 13 kb)

Additional file 8: Figure S4. Quantile-quantile (QQ) plot for differential exon usage between current and former smokers using the topSplice exon-based T-statistic. (PNG 16 kb)

Additional file 9: Figure S5. Exon-level expression of *EPS15*. The top plot shows mean normalized counts by smoking status on the log scale for each analyzed exon. One exon showed significant differential usage (ENSE00001810132). The bottom table maps tested exons to known transcripts (1 = exon present in that transcript, 0 = exon not present in that transcript). (PNG 92 kb)

Additional file 10: Figure S6. Exon-level expression of *SASH1*. The top plot shows mean normalized counts by smoking status on the log scale for each analyzed exon. One exon showed significant differential usage (ENSE00001444573). The bottom table maps tested exons to known transcripts (1 = exon present in that transcript, 0 = exon not present in that transcript). (PNG 64 kb)

Additional file 11: Figure S7. Exon-level expression of *AREL1*. The top plot shows mean normalized counts by smoking status on the log scale for each analyzed exon. One exon showed significant differential usage (ENSE00001400828). The bottom table maps tested exons to known transcripts (1 = exon present in that transcript, 0 = exon not present in that transcript). (PNG 102 kb)

Additional file 12: Figure S8. Exon-level expression of last 11 exons of *UTRN*. The top plot shows mean normalized counts on the log scale for each exon passing filtering by smoking status. There was one exon that showed significant differential usage between current and former smokers (ENSE00001444981). The bottom table maps tested exons to known transcripts (1 = exon present in that transcript, 0 = exon not present in that transcript). (PNG 141 kb)

Additional file 13: Figure S9. Exon-level expression of *MAN1A2.* The top plot shows mean normalized counts by smoking status on the log scale for each analyzed exon. One exon that showed significant differential usage (ENSE00001635177). The bottom table maps tested exons to known transcripts (1 = exon present in that transcript, 0 = exon not present in that transcript). (PNG 51 kb)

Additional file 14: Figure S10. Exon-level expression of *LRRN3.* The top plot shows mean normalized counts by smoking status on the log scale for each analyzed exon. One exon showed significant differential usage between current and former smokers (ENSE00001231507). The bottom table maps tested exons to known transcripts (1 = exon present in that transcript, 0 = exon not present in that transcript). (PNG 46 kb)

Additional file 15: Figure S11. Exon-level expression of *ERAP1.* The top plot shows mean normalized counts by smoking status on the log scale for each analyzed exon. Two exons showed significant differential usage between current and former smokers (ENSE00001175333, ENSE00001641703). The bottom table maps tested exons to known transcripts (1 = exon present in that transcript, 0 = exon not present in that transcript). (PNG 71 kb)

Additional file 16: IRB approval and complete list of acknowledgements. (DOCX 20 kb)

## Abbreviations

CBC: Complete blood cell count
DEU: Differential exon usage
lncRNA: Long non-coding RNA
RNA-seq: RNA sequencing
