# Correction to: RNA sequencing identifies novel non-coding RNA and exon-specific effects associated with cigarette smoking

**DOI:** 10.1186/s12920-019-0617-1

**Published:** 2019-11-18

**Authors:** Margaret M. Parker, Robert P. Chase, Andrew Lamb, Alejandro Reyes, Aabida Saferali, Jeong H. Yun, Blanca E. Himes, Edwin K. Silverman, Craig P. Hersh, Peter J. Castaldi

**Affiliations:** 1 Channing Division of Network Medicine, Brigham and Women’s Hospital, 181 Longwood Ave, Boston, MA USA; 2 Harvard Medical School, Boston, MA 02115 USA; 3 Department of Biostatistics and Computational Biology, Dana-Farber Cancer Institute, Boston, MA USA; 4 Division of Pulmonary and Critical Care Medicine, Brigham and Women’s Hospital, Boston, MA USA; 5 Department of Biostatistics, Epidemiology, and Informatics, Perelman School of Medicine, University of Pennsylvania, Philadelphia, PA USA; 6 Division of General Internal Medicine and Primary Care, Brigham and Women’s Hospital, Boston, MA USA

**Correction to: BMC Med Genomics (2017) 10:58**

**https://doi.org/10.1186/s12920-017-0295-9**

Following publication of the original article [1], the authors provided an updated accession number in the “Availability of data and materials” section of the declarations. The accession number is listed as GSE9753, but the correct number is GSE97531.
