# Supplementary figures and images for: RNA sequencing identifies novel non-coding RNA and exon-specific effects associated with cigarette smoking
Source: BMC Med Genomics. 2017 Oct 6;10:58. doi: 10.1186/s12920-017-0295-9 (PMC6225866; doi:10.1186/s12920-017-0295-9)

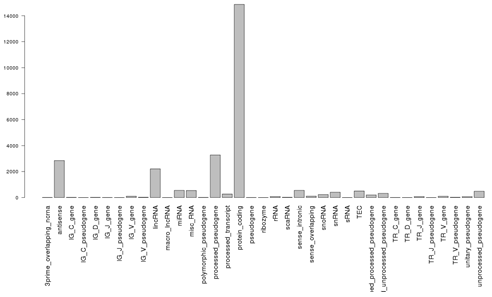

Supplement: Supplementary file 1 — Gene annotation of 27,885 observed genes (Ensembl version 81 annotation). (PNG 19 kb) [file 12920_2017_295_MOESM1_ESM.png]

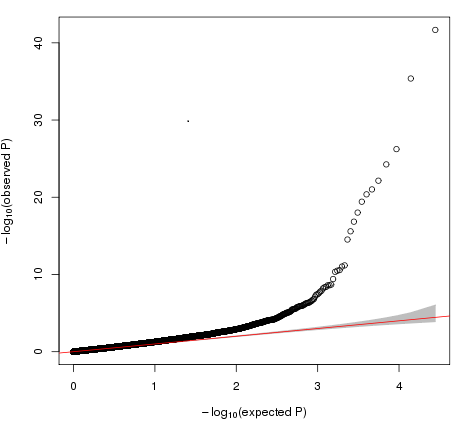

Supplement: Supplementary file 4 — Quantile-quantile (QQ) plots for differential gene expression analysis between current and former smokers using voom/limma. (PNG 14 kb) [file 12920_2017_295_MOESM4_ESM.png]

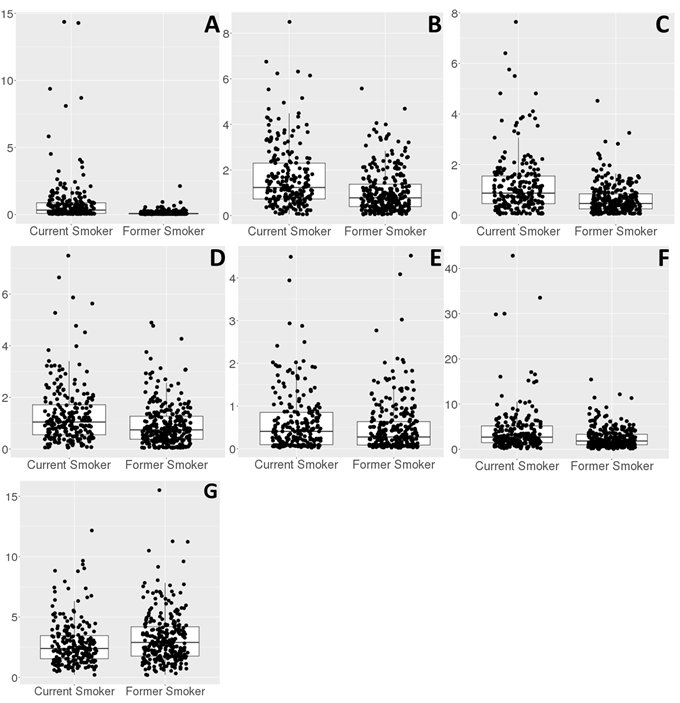

Supplement: Supplementary file 5 — Normalized counts in current versus former smokers for lncRNAs that are significantly differentially expressed: A) ENSG00000253230; B) ENSG00000227240; C) ENSG00000230817; D) ENSG00000237011; E) ENSG00000267453; F) ENSG00000254275; G) ENSG00000227508. In 6 of 7 differentially expressed lncRNAs, current smokers have higher expression than former smokers. Y-axis represents log2 expression level. (PNG 118 kb) [file 12920_2017_295_MOESM5_ESM.png]

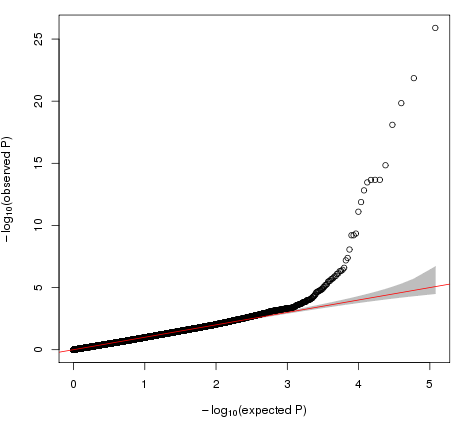

Supplement: Supplementary file 8 — Quantile-quantile (QQ) plot for differential exon usage between current and former smokers using the topSplice exon-based T-statistic. (PNG 16 kb) [file 12920_2017_295_MOESM8_ESM.png]

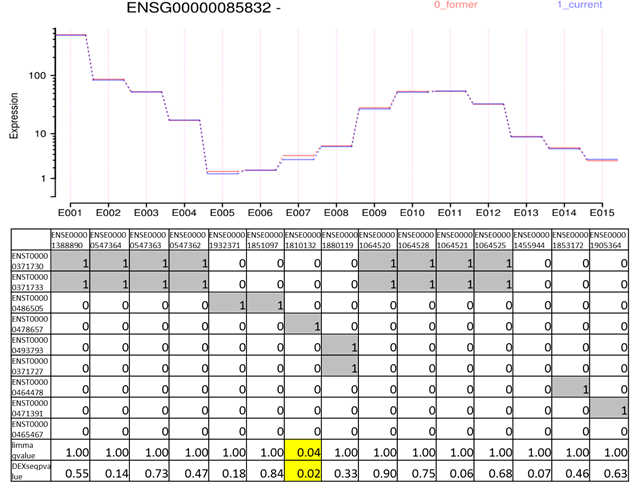

Supplement: Supplementary file 9 — Exon-level expression of EPS15. The top plot shows mean normalized counts by smoking status on the log scale for each analyzed exon. One exon showed significant differential usage (ENSE00001810132). The bottom table maps tested exons to known transcripts (1 = exon present in that transcript, 0 = exon not present in that transcript). (PNG 92 kb) [file 12920_2017_295_MOESM9_ESM.png]

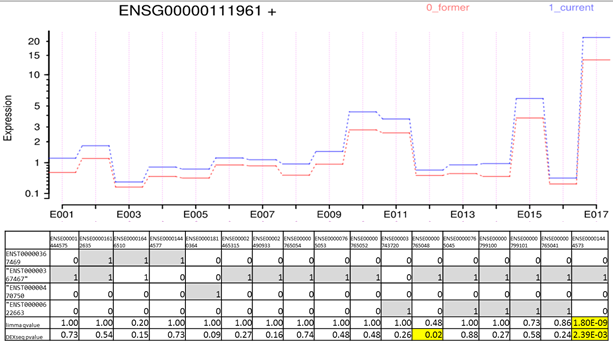

Supplement: Supplementary file 10 — Exon-level expression of SASH1. The top plot shows mean normalized counts by smoking status on the log scale for each analyzed exon. One exon showed significant differential usage (ENSE00001444573). The bottom table maps tested exons to known transcripts (1 = exon present in that transcript, 0 = exon not present in that transcript). (PNG 64 kb) [file 12920_2017_295_MOESM10_ESM.png]

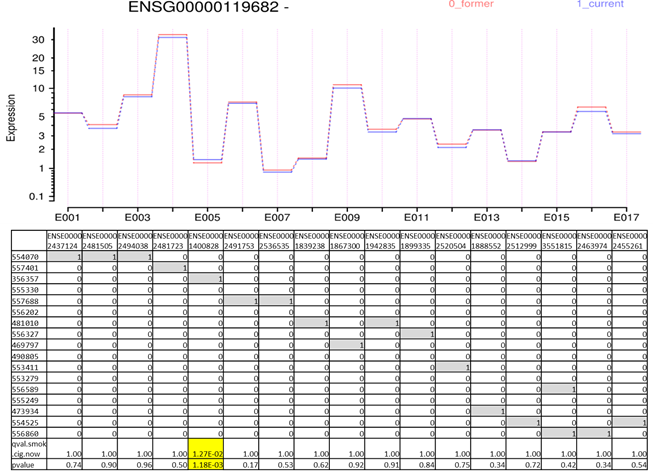

Supplement: Supplementary file 11 — Exon-level expression of AREL1. The top plot shows mean normalized counts by smoking status on the log scale for each analyzed exon. One exon showed significant differential usage (ENSE00001400828). The bottom table maps tested exons to known transcripts (1 = exon present in that transcript, 0 = exon not present in that transcript). (PNG 102 kb) [file 12920_2017_295_MOESM11_ESM.png]

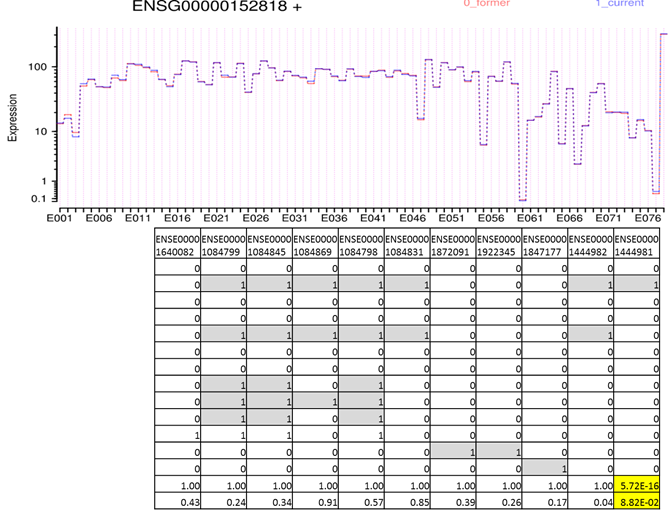

Supplement: Supplementary file 12 — Exon-level expression of last 11 exons of UTRN. The top plot shows mean normalized counts on the log scale for each exon passing filtering by smoking status. There was one exon that showed significant differential usage between current and former smokers (ENSE00001444981). The bottom table maps tested exons to known transcripts (1 = exon present in that transcript, 0 = exon not present in that transcript). (PNG 141 kb) [file 12920_2017_295_MOESM12_ESM.png]

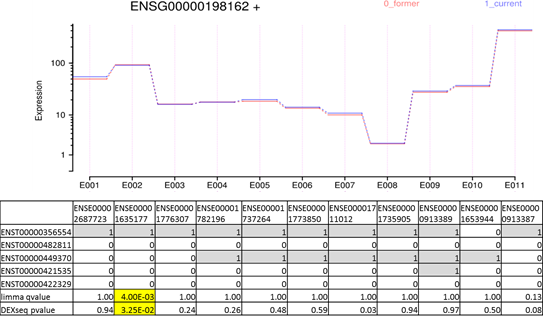

Supplement: Supplementary file 13 — Exon-level expression of MAN1A2. The top plot shows mean normalized counts by smoking status on the log scale for each analyzed exon. One exon that showed significant differential usage (ENSE00001635177). The bottom table maps tested exons to known transcripts (1 = exon present in that transcript, 0 = exon not present in that transcript). (PNG 51 kb) [file 12920_2017_295_MOESM13_ESM.png]

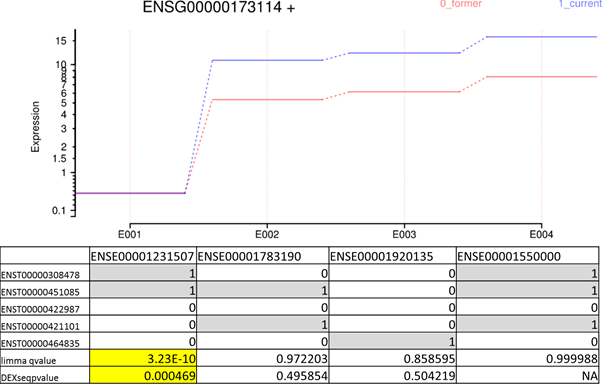

Supplement: Supplementary file 14 — Exon-level expression of LRRN3. The top plot shows mean normalized counts by smoking status on the log scale for each analyzed exon. One exon showed significant differential usage between current and former smokers (ENSE00001231507). The bottom table maps tested exons to known transcripts (1 = exon present in that transcript, 0 = exon not present in that transcript). (PNG 46 kb) [file 12920_2017_295_MOESM14_ESM.png]

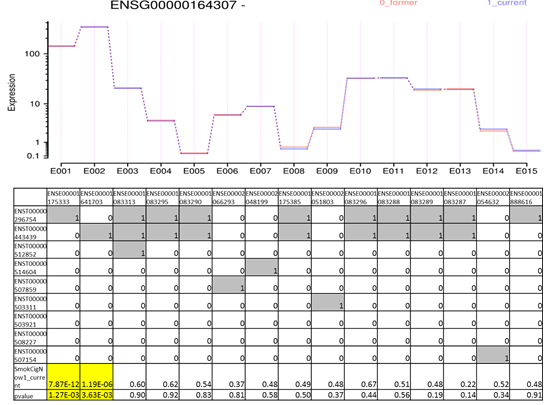

Supplement: Supplementary file 15 — Exon-level expression of ERAP1. The top plot shows mean normalized counts by smoking status on the log scale for each analyzed exon. Two exons showed significant differential usage between current and former smokers (ENSE00001175333, ENSE00001641703). The bottom table maps tested exons to known transcripts (1 = exon present in that transcript, 0 = exon not present in that transcript). (PNG 71 kb) [file 12920_2017_295_MOESM15_ESM.png]
